# Association of serum total 25-hydroxy-vitamin D concentration and risk of all-cause, cardiovascular and malignancies-specific mortality in patients with hyperlipidemia in the United States

**DOI:** 10.3389/fnut.2022.971720

**Published:** 2022-10-20

**Authors:** Xueqin Chen, Mingge Zhou, Hui Yan, Jiatian Chen, Yuetao Wang, Xiaofei Mo

**Affiliations:** ^1^Department of Nuclear Medicine, The Third Affiliated Hospital of Soochow University, Changzhou, China; ^2^Changzhou Key Laboratory of Molecular Imaging, Changzhou, China

**Keywords:** vitamin D, hyperlipidemia, NHANES, cardiovascular mortality, all cause mortality

## Abstract

**Background:**

Vitamin D (VD) plays an important role in decreasing the risk of adverse events for various metabolic diseases. However, for patients with hyperlipidemia, the relationship between the main VD storage within the body known as serum 25-hydroxy-VD [25(OH)VD] and the risk of all-cause, cardiovascular and malignancies-specific mortality is still unclear.

**Materials and methods:**

A total of 6740 participants above the age of 20 years with hyperlipidemia who completed the National Health and Nutrition Examination Survey (NHANES) between 2007 and 2016 and were followed up until 2019 were included in the study. The weighted Cox proportional hazards regression model and weighted competing risk regression model were used to evaluate the risk for all-cause, cardiovascular and malignancy-related mortality in relation to the serum 25(OH)VD. The model was adjusted according to age, gender, race, body mass index, lipids status, medication usage, the Charlson comorbidity index and healthy eating index. The last restricted cubic spline (RCS) method was used to present the relationship between hazard ratios (HR) associated with diverse cause-specified modalities and the serum 25(OH)VD levels.

**Results:**

Serum 25(OH)VD was identified as an independent factor for mortality. Lower serum 25(OH)VD under the threshold of 25.6 and 25.2 ng/ml were significantly associated with a higher risk for all-cause and cardiovascular mortalities, respectively. However, no association was found between malignancy-specific mortality and serum 25(OH)VD.

**Conclusion:**

Serum 25(OH)VD were identified as an independent factor associated with risks of all-cause and cardiovascular mortalities in patient with hyperlipidemia. Moreover, lower serum 25(OH)VD than 25.6 and 25.2 ng/mL were, respectively, associated with a gradual increase in a risk for all-cause and cardiovascular mortality in patients with hyperlipidemia, and therefore regular monitoring of VD levels and correction of VD deficiency is recommended in those patients.

## Introduction

Hyperlipidemia (HL) is a common metabolic disease characterized by dysregulation of low-density lipoprotein cholesterol (LDL-C), high-density lipoprotein cholesterol (HDL-C), triacylglycerol (TG), and cholesterol (CHL). Over 100 million people were diagnosed with hypercholesterolemia (total CHL levels > 240 mg/dL) in 2017, and another 31 million adults were diagnosed with elevated LDL-C levels in the United States (1). HL can reduce the patients’ quality of life and increases their risk of developing cardiovascular disease by 2 to 3 times (2). Therefore, there is a need to develop treatment strategies to control blood lipid levels in patients with HL to improve their survival.

Studies have shown that several nutrients, such as vitamin D (VD), may have an important role in lowering lipid levels and in reducing the risk of mortality (3), especially in patients with hypertension (4) and type-2 diabetes (5). Therefore, VD supplements are often prescribed to patients with metabolic diseases suffering from VD deficiency. VD is stored in the body as 25-hydroxy-vitamin D [25(OH)VD], which encompasses both 25(OH)VD3 and 25(OH)VD2 forms (6). Because of its stable nature and long half-life, serum 25(OH)VD could be used as an optimal indicator for monitoring of VD levels. However, it is still debatable whether the administration of VD supplements could improve hypertension (7), insulin sensitivity (8), or lipid parameters (9). Therefore, the benefit of VD supplements on metabolic disorders is still unclear. In addition, relatively few studies evaluated the relationship between serum 25(OH)VD and the risk of mortality in patients with HL and VD deficiency. Furthermore, numerous factors can affect the normal VD levels, including age, diet, nutritional status, and underlying comorbidities. However, these factors have not been totally taken into account in current risk survival models.

Therefore, this study aimed to analyze the association between serum 25(OH)VD and the risk of all-cause and disease-specific (cardiovascular and malignancy) mortality in patients with HL in the United States (US) to guide the use of VD supplements in patients with HL.

## Materials and methods

### Data collection

The data was obtained from the National Health and Nutrition Examination database (NHANES). The NHANES was a national survey conducted by the National Center for Health Statistics to assess the health and nutritional status of adults and children in the US. The survey collected data on participants’ demographics, socioeconomic status, diet, general health, medical examinations, and laboratory tests.

Participants above the age of 20 years with HL who completed the National Health and Nutrition Examination Survey (NHANES) between 2007 and 2016 and were followed up until 2019 were included in the study. HL was defined as a low-density lipoprotein-cholesterol (LDL-C) ≥ 130mg/dL (3.37 mmol/L), triglyceride (TG) ≥ 150mg/dL (1.7 mmol/L), total cholesterol (TC) ≥ 200 mg/dL (5.18mmol/L) or high-density lipoprotein-cholesterol (HDL-C) < 40mg/dL (1.04mmol/L) in males and 50 mg/dL (1.30mmol/L) in females (10). The participants’ demographic information, including age at participating in the survey, ethnicity, gender, and body mass index (BMI), were extracted from the survey. Moreover, serum 25(OH)VD level, daily VD intake, comorbidities, and medication usage (in the past 30 days) were also extracted. The Charlson comorbidity index (CCI) were calculated according to questionnaire survey and examination, the healthy eating index (HEI) were calculated according to the HEI-2015 guidelines (11). Daily VD intake and dietary nutrients intake were calculated the average values using the data obtained from two 24h dietary recall interviews.

**FIGURE 1:**
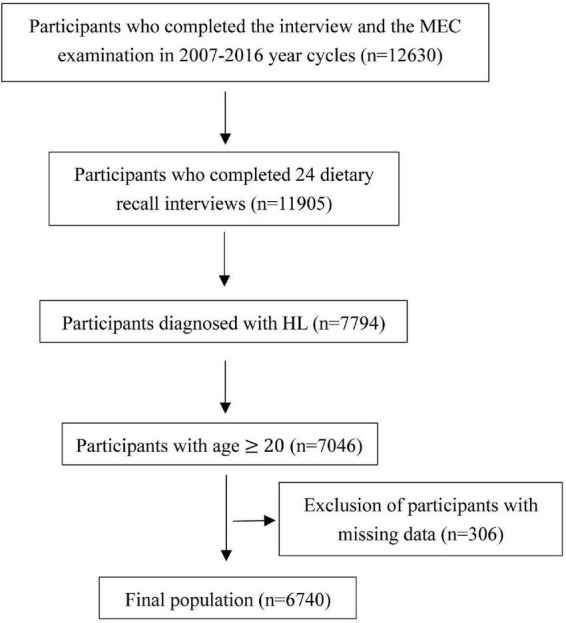
Flowchart of participants enrollment.

The follow-up survival data were obtained from the National Center for Health Statistics. The International Classification of Diseases version 10 (ICD-10) was used to classify the causes of death into cardiovascular disease (ICD CODES I00-I09, I11, I13, I20-I51, and I60-I69) and malignancy-related (ICD coded C00-C97). Collection of data was shown in Figure 1.

### Measurement methods of blood indicators

The serum 25(OH)VD was defined as the sum of serum 25(OH)D2, 25(OH)D3, and epi-25(OH)D3. All the measurements were acquired using ultra-high-performance liquid chromatography-tandem mass spectrometry (UHPLC-MS/MS). The serum levels of HDL-C, TC, and TG were tested using the Roche Modular P Chemistry Analyzer for enzymatic assays (12). LDL-C is calculated from measured values of TC, TG, and HDL-C according to the Friedewald calculation: [LDL-C] = [TC] – [HDL-C] – [TG/5] (13), where all values are expressed in mg/dL. The calculation is valid for TG levels less than or equal to 400 mg/dL, so only for subjects with TG levels lower of 400 mg/dl, were the data for LDL-C provided in the NHANES datasets.

### Statistical analysis

Vitamin D status was defined as “deficient” [serum 25(OH)VD < 20 ng/ml], “insufficient” [serum 25(OH)VD = 20–30 ng/ml] and “sufficient” [serum 25(OH)VD > 30 ng/ml] (14). The serum 25(OH)VD levels were divided into five groups by the quintile values. The participants’ age, race, gender, BMI, CCI, HEI, lipid status, the length of follow-up and medication usage were treated as covariates. Moreover, BMI was categorized into 3 groups: <25 (normal), 25–29.9 (overweight) and ≥30.0 (obesity) (15); HEI was categorized into 3 groups: <50.0 (poor dietary), 50.0–79.9 (need to improve) and ≥80.0 (healthy dietary) (16); VD intake was also categorized into 3 groups: <10 μg/d (less than the US Estimated Average Requirement, EAR), 10–15 μg/d and >15 μg/d (above the US Recommended Dietary Allowance, RDA) (17, 18). The continuous data were evaluated for normality. The association between continuous variables was calculated using the analysis of variance (ANOVA), and the Chi-square test was used to compare the categorical variables. The Pearson’s correlation coefficient was used to calculate the correlation between the normally distributed continuous variables and the Spearman’s correlation coefficient was used to calculate the correlation between the categorical variables and non-normally distributed continuous variables.

The risk for all cause-specific mortality in relation to the serum 25(OH)VD was estimated by the Cox proportional hazard (COXPH) model. For the malignancy/cardiovascular-specific mortalities, the competing risk regression (CRR) model was used to estimate the hazard ratios (HRs) and their 95% confidence intervals (95% CI). The sample weight was taken into account in the above models. The time dependent receiver operation curves (ROC) were calculated, then area under curve (AUC) of ROC by each time point were depicted for examining discriminative abilities of models, a bigger AUC curve indicated higher discriminative ability (19). Moreover, calibration curves were also depicted for tested models, the curves more closed to diagonal suggested the better calibration of models. Finally, the restricted cubic splines (RCS) with the unweighted Cox proportional hazard models were depicted to directly present the dose-response associations between serum 25(OH)VD and HRs of all cause and malignancy/cardiovascular-specific mortalities. Apart from including the length of follow-up, the models were adjusted for mortality covariates, including age, gender, race, the HEI and CCI scores, lipid status and medication usage. Since mortality in our study was a rare event, the Poisson’s regression model was also used to estimate the incidence-rate mortality ratio. A two-sided *p*-value below 0.05 was considered statistically significant. The data were analyzed using the R software (version:4.2.1). The COX PHM was performed using the “rms” R package, and the Poisson’s regression model was performed by the “lms” R package. The time-AUC of ROC and calibration curves were calculated using the “riskRegression” R package, The RCS model was built using the “survminer” R package, and the Pearson’s correlation coefficient map was plotted *via* the “corrplot” R package.

## Results

### Characteristics of the participants

A total of 6740 participants were included in the study. The characteristics of the participants are summarized in Table 1. The included participants were 20-80 years old. Both genders were quite equally represented, and majority of the participants were non-Hispanic whites (46.8%). Almost 4/5 of the included participants were overweight or obese (77.1%). Almost half of the participants had a CCI score of 0 (45.2%), while based on the HEI scores, only small percentage of the participants (3.4%) were classified as healthy with an HEI score above 80. Only less than a third of the participants (29.8%) have received hypolipidemic medication treatment, such as statins (91.7%), fenofibrate (2.0%) and gemfibrozil (1.8%). The mean serum 25(OH)VD in the whole sample was 26.7 ng/mL, while the serum 25(OH)VD quintile levels were at 17.3, 23.2, 28.3, and 35.0 ng/mL. Only about 1/3 of the participants (34.7%) had VD sufficient status, while VD insufficiency and deficiency were detected in 36.6% and 28.6% of the participants, respectively. The non-Hispanic whites had the highest VD levels, while non-Hispanic Blacks and Mexican Americans had the lowest levels, with mean values of 30.6 ± 0.6, 21.1 ± 0.5 and 22.8 ± 0.4 ng/mL, respectively (*p* < 0.01). Obese people had the lowest vitamin D levels, compared with normal weight and overweight with mean values of 24.6 ± 0.4, 29.6 ± 0.4, and 27.4 ± 0.4 ng/mL, respectively (*p* < 0.01). The mean and median values of participants’ daily VD intake were 4.7 ± 1.0 and 3.6 μg/d, respectively. The daily VD intake was less than the EAR (10 μg/d) in most of the participants (91.2%), while only 3.1% of the participants had intake above the RDA (15 μg/d). Participants with highest VD levels (higher quintiles) had higher HDL-C and lower TG levels. In general, with increase of serum 25(OH)VD, there was a trend for increased HDL-C and TC, and decreased TG and LDL-C levels; there was also a trend for increased HEI score, VD intake, and decreased BMI (*p* < 0.01). During the 51372 person-years of follow-up, 752 deaths were recorded (overall, in 11.2% of participants), of which 41.9% were due to cardiovascular disease, 31.3% were caused by malignancies, and 26.9% were attributed to other factors.

**TABLE 1:** The baseline of participants with hyperlipidemia according to serum 25(OH)VD.

	Serum 25(OH)VD (ng/mL)						
	Total	<17.3	17.3–23.1	23.2–28.2	28.3–35.0	>35.0	*p*-value
Number of cases	6740	1343 (19.9)	1353 (20.0)	1352 (20.1)	1344 (19.9)	1348 (20.0)	
Age	53.1 (0.2)	48.3 (0.4)	49.5 (0.4)	52.4 (0.4)	55.0 (0.4)	60.6 (0.4)	
20–40	1787 (26.5)	481 (35.8)	463 (34.2)	362 (26.8)	299 (22.3)	182 (13.5)	<0.01
41–60	2482 (36.8)	513 (38.2)	515 (38.1)	517 (38.2)	507 (37.7)	430 (31.9)	
> 61	2471 (36.7)	349 (26.0)	375 (27.7)	473 (35.0)	538 (40.0)	736 (54.6)	
**Gender**							
Female	3994 (53.0)	808 (53.8)	772 (51.3)	729 (48.1)	756 (50.2)	929 (61.8)	<0.01
Male	3539 (47.0)	695 (46.2)	732 (48.7)	788 (51.9)	749 (49.8)	575 (38.2)	
**Race/ethnicity**							
Non-Hispanic White	3119 (46.3)	296 (22.0)	453 (33.5)	623 (46.1)	817 (60.8)	930 (69.0)	<0.01
Non-Hispanic Black	1192 (17.7)	504 (37.5)	257 (19.0)	189 (14.0)	121 (9.0)	121 (9.0)	
Mexican American	1017 (15.1)	279 (20.8)	280 (28.2)	233 (17.2)	146 (10.9)	79 (5.9)	
Others^1^	1412 (21.0)	264 (19.7)	363 (26.8)	307 (22.7)	260 (19.4)	218 (16.2)	
BMI	30.0 (0.2)	32.1 (0.3)	30.9 (0.2)	29.7 (0.2)	29.3 (0.2)	28.1 (0.2)	
<25.0	1544 (22.9)	234 (17.4)	220 (16.3)	307 (22.7)	344 (25.6)	439 (32.6)	<0.01
25.0–29.9	2297 (34.1)	373 (27.8)	478 (35.3)	461 (34.1)	488 (36.3)	497 (36.9)	
=30.0	2899 (43.0)	736 (54.8)	655 (48.4)	584 (43.2)	512 (38.1)	412 (30.6)	
CCI	1.2 (1.4)	1.1 (1.4)	1.2 (1.3)	1.3 (1.3)	1.6 (1.1)	1.3 (1.3)	
0	3046 (45.2)	685 (51.0)	684 (50.5)	630 (46.6)	591 (44.0)	456 (33.8)	<0.01
1	1465 (21.7)	258 (19.2)	309 (22.8)	284 (21.0)	288 (21.4)	326 (24.2)	
2	909 (13.5)	172 (12.8)	159 (11.8)	189 (14.0)	182 (13.5)	207 (15.4)	
=3	1320 (19.6)	228 (17.0)	201 (14.9)	249 (18.4)	283 (21.1)	359 (26.6)	
HDL (mg/dL)	52.4 (0.2)	50.1 (0.4)	49.3 (0.4)	50.6 (0.4)	53.6 (0.5)	58.4 (0.5)	<0.01
LDL (mg/dL)	121.8 (0.5)	123.6 (1.0)	123.0 (1.0)	121.9 (1.0)	121.3 (1.0)	119.2 (1.0)	<0.01
TG (mg/dL)	133.1 (0.8)	133.1 (1.9)	134.3 (1.9)	135.7 (1.8)	133.5 (1.8)	129.1 (1.7)	<0.01
TC (mg/dL)	200.8 (0.5)	200.2 (1.2)	199.3 (1.2)	199.6 (1.2)	201.6 (1.2)	203.4 (1.2)	<0.01
Healthy eatig index	54.5 (0.2)	50.9 (0.3)	53.4 (0.2)	54.8 (0.2)	55.7 (0.2)	57.6 (0.2)	
<50.0	2613 (38.8)	659 (49.1)	557 (41.2)	510 (37.7)	484 (36.0)	403 (29.9)	<0.01
50.0–79.9	3899 (57.9)	661 (49.2)	759 (56.1)	794 (58.7)	811 (60.3)	874 (64.8)	
=80.0	228 (3.4)	23 (1.7)	37 (2.7)	48 (3.6)	49 (3.7)	71 (5.3)	
Vitamin D intake (μg/d)	4.7 (1.0)	3.6 (0.9)	4.5 (1.0)	5.1 (0.9)	5.2 (1.0)	5.0 (0.9)	
Median	3.6	2.7	3.4	4.0	4.0	3.8	
<10	6146 (91.2)	1289 (96.0)	1246 (92.1)	1203 (89.0)	1193 (88.8)	1215 (90.1)	<0.01
10–15	384 (5.7)	34 (2.5)	71 (5.3)	98 (7.3)	102 (7.6)	79 (5.9)	
>15	210 (3.1)	20 (1.5)	36 (2.7)	51 (3.8)	49 (3.7)	54 (4.0)	
Medication usage							<0.01
No	2378 (35.3)	633 (47.1)	589 (43.5)	512 (37.9)	398 (29.6)	246 (18.3)	
hypolipidemic medications	2010 (29.8)	291 (21.7)	310 (22.9)	383 (28.3)	449 (33.4)	577 (42.8)	
Others^2^	2352 (34.9)	419 (31.2)	454 (33.6)	457 (33.8)	497 (37.0)	525 (39.0)	
Outcomes							<0.01
Alive	5988 (88.8)	1189 (88.5)	1226 (90.6)	1201 (88.8)	1200 (89.3)	1172 (86.9)	
Cardiovascular mortality	315 (4.7)	68 (5.1)	62 (4.6)	71 (5.3)	52 (3.9)	62 (4.6)	
Malignancies-specified mortality	235 (3.5)	53 (4.0)	32 (2.4)	48 (3.6)	48 (3.6)	54 (4.0)	
Other causes	202 (3.0)	33 (2.5)	33 (2.4)	32 (2.4)	44 (3.3)	60 (4.5)	
Length of follow-up							<0.01
7–12 years	4206 (62.4)	872 (64.9)	867 (64.1)	882 (65.2)	840 (62.5)	745 (17.7)	
3–6 years	2534 (37.6)	471 (35.1)	486 (35.9)	470 (34.8)	504 (37.5)	603 (23.8)	

Measurement data were as recorded means (SE) and count data were numbers (percentage); 1, Other races indicated Multi-Racial population and Hispanics; 2, indicated drugs except hypolipidemic medications. CCI, Charlson Comorbidity Index; HEI, healthy eating index; BMI, body mass index; TC, total cholesterol; TG, triglyceride; HDL-C, high-density lipoprotein-cholesterol; LDL-C, low-density lipoprotein-cholesterol.

### Correlations between variables in the present study

The results of the Pearson’s and Spearman’s inter-correlations between the included variables are summarized in Table 2. In summary, serum 25(OH)VD positively correlated with age, Non-Hispanic White race, CCI score, HEI score, HDL-C levels, TC levels, hypolipidemic drugs usage, while negatively correlated with male sex, other races except Non-Hispanic White, BMI, LDL-C and TG levels with statistical significance (Table 2). In regression models, the variance inflation factors between these variables were also calculated, and were less than 3 for all variables included: serum 25(OH)VD (1.28), gender (1.53), age (1.43), Non-Hispanic White (2.74), Non-Hispanic Black (2.55), other races (2.03) BMI (1.13), CCI (1.33), HEI (1.10), medication usage (1.15), HDL-C (1.81), LDL-C (1.87), TG (1.15), TC (2.04) and the length of follow-up (1.02), respectively. These findings suggest that the variables in the present study were independent, so multicollinearity was not an issue.

**TABLE 2:** The correlation coefficients between variables in the present study.

	Serum 25 (OH)VD	Male	Age	Non-Hispanic White	Non-Hispanic Black	Mexican American	Other races^1^	CCI	BMI	hypolipidemic medication usage	HEI	HDL-C	LDL-C	TG	TC	Length of follow-up
Serum 25(OH)VD	1.000	**−0**.**046**	**0**.**259**	**0**.**353**	**−0**.**263**	**−0**.**156**	**−0**.**049**	**0**.**114**	**−0**.**203**	**0**.**153**	**0**.**137**	**0**.**212**	**−0**.**033**	**−0**.**027**	**0**.**045**	**0**.**065**
Male	**−0**.**046**	1.000	-0.001	0.023	**−0**.**035**	-0.003	0.007	0.009	**−0**.**077**	**−0**.**127**	**−0**.**099**	**−0**.**290**	**−0**.**030**	**0**.**109**	**−0**.**103**	-0.007
Age	**0**.**259**	-0.001	1.000	**0**.**187**	**−0**.**038**	**−0**.**128**	**−0**.**081**	**0**.**431**	**−0**.**072**	**0**.**265**	**0**.**179**	**0**.**189**	**−0**.**129**	**−0**.**031**	**−0**.**049**	**0**.**031**
Non-Hispanic White	**0**.**353**	0.023	**0**.**187**	1.000	**−0**.**430**	**−0**.**391**	**−0**.**478**	**0**.**137**	**−0**.**041**	**0**.**159**	**−0**.**060**	0.020	**−0**.**052**	**0**.**041**	**−0**.**024**	**−0**.**042**
Non-Hispanic Black	**−0**.**263**	**−0**.**035**	**−0**.**038**	**−0**.**430**	1.000	**−0**.**195**	**−0**.**239**	-0.027	0.141	**0**.**023**	**−0**.**030**	**0**.**079**	**0**.**031**	**−0**.**187**	-0.002	0.014
Mexican American	**−0**.**156**	-0.003	**−0**.**128**	**−0**.**391**	**−0**.**195**	1.000	**−0**.**217**	**−0**.**076**	**0**.**049**	**−0**.**139**	-0.005	**−0**.**080**	0.009	**0**.**091**	**0**.**005**	**−0**.**029**
Other races^1^	**−0**.**049**	0.007	**−0**.**081**	**−0**.**478**	**−0**.**239**	**−0**.**217**	1.000	**−0**.**076**	**−0**.**124**	**−0**.**093**	**0**.**106**	-0.028	0.028	**0**.**045**	**0**.**027**	**0**.**064**
CCI	**0**.**114**	0.009	**0**.**431**	**0**.**137**	**−0**.**027**	**−0**.**076**	**−0**.**076**	1.000	**0**.**118**	**0**.**276**	**0**.**008**	-0.030	-0.175	**0**.**062**	-0.143	**0**.**036**
BMI	**−0**.**203**	**−0**.**077**	**−0**.**072**	**−0**.**041**	**0**.**141**	**0**.**049**	**−0**.**124**	**0**.**118**	1.000	**0**.**064**	**−0**.**112**	-0.257	-0.042	**0**.**127**	**−0**.**095**	**0**.**028**
hypolipidemic medication usage	**0**.**153**	**−0**.**127**	**0**.**265**	**0**.**159**	0.023	**−0**.**139**	**−0**.**093**	**0**.**276**	**0**.**064**	1.000	**0**.**035**	0.079	-0.009	**0**.**033**	**0**.**033**	0.004
HEI	**0**.**137**	**−0**.**099**	**0**.**179**	**−0**.**060**	**−0**.**030**	-0.005	**0**.**106**	**0**.**008**	**−0**.**112**	**0**.**035**	1.000	0.141	-0.023	**−0**.**061**	0.015	**−0**.**027**
HDL-C	**0**.**212**	**−0**.**290**	**0**.**189**	0.020	**0**.**079**	**−0**.**080**	-0.028	-0.030	-0.257	0.079	0.141	1.000	0.113	**−0**.**394**	**0**.**358**	0.012
LDL-C	**−0**.**033**	**−0**.**030**	**−0**.**129**	**−0**.**052**	**0**.**031**	0.009	0.028	-0.175	-0.042	-0.009	-0.023	0.113	1.000	**0**.**046**	**0**.**923**	**−0**.**043**
TG	**−0**.**027**	**0**.**109**	**−0**.**031**	**0**.**041**	**−0**.**187**	**0**.**091**	**0**.**045**	**0**.**062**	**0**.**127**	**0**.**033**	**−0**.**061**	**−0**.**394**	**0**.**046**	1.000	**0**.**205**	**−0**.**106**
TC	**0**.**045**	**−0**.**103**	**−0**.**049**	**−0**.**024**	-0.002	**0**.**005**	**0**.**027**	**−0**.**143**	**−0**.**095**	**0**.**033**	0.015	**0**.**358**	**0**.**923**	**0**.**205**	1.000	-0.054
Length of follow-up	**0**.**065**	-0.007	**0**.**031**	**−0**.**042**	0.014	**−0**.**029**	**0**.**064**	**0**.**036**	**0**.**028**	0.004	**−0**.**027**	0.012	**−0**.**043**	**−0**.**106**	-0.054	1.000

The correlations among continuous variables were calculated the Pearson correlation coefficients and categorical variables were calculated the Spearman correlation coefficients; 1, other races indicated Multi-Racial population and Hispanics; Bold font means *p* < 0.05. CCI, Charlson Comorbidity Index; HEI, healthy eating index; BMI, body mass index; TC, total cholesterol; TG, triglyceride; HDL-C, high-density lipoprotein-cholesterol; LDL-C, low-density lipoprotein-cholesterol.

### Association between serum 25-hydroxy-vitamin D with mortality risk for patients with hyperlipidemia

**FIGURE 2:**
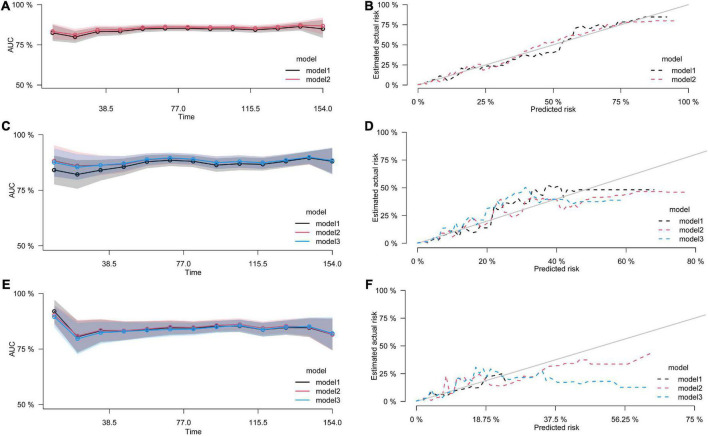
The discrimination and calibration curves of predict models. **(A,B)** All-cause mortality; **(C,D)** cardiovascular mortality; **(E,F)** malignancies-specific mortality. The ribbons indicate the 95% CIs of AUC.

As shown in Table 3, serum 25(OH)VD levels were significantly associated with all-cause and cardiovascular-specific mortality in participants with HL. After fully adjusting the COXPH and CRR models for the potential confounders, the risk for all-cause mortality significantly increased with 25(OH)D levels below 23.1 ng/mL (model 2), while the risk for cardiovascular mortality significantly increased with 25(OH)D levels below 17.3 ng/mL (model 3). The risk for overall mortality increased in the first 2 quintiles, with gradual change, and then stabilized, while the risk for cardiovascular mortality significantly increased only in the first quintile. However, no significant association was noted between serum 25(OH)VD and the risk of malignancies-related mortality. The results were still robust after multivariable- adjusted estimation by different regression models. All models were tested their discriminative and calibration abilities, and the fully adjusted COXPH and CRR models presented better predictive performance (Figure 2).

**TABLE 3:** Association between different levels of 25(OH)VD with risk of all-causes, cardiovascular diseases, and malignancy related mortalities in patients with HL.

		Serum 25(OH)VD (ng/mL)			
	>35.0	<17.3	17.3–23.1	23.2–28.2	28.3–35.0
**All-cause mortality**					
Model 1	1.00	**2.18 (1.62–2.95)**	**1.70 (1.28–2.25)**	1.25 (0.96**–**1.63)	0.98 (0.75**–**1.28)
Model 2	1.00	**2.06 (1.51–2.82)**	**1.65 (1.22–2.22)**	1.19 (0.91**–**1.56)	0.95 (0.72**–**1.25)
**Cardiovascular mortality**					
Model 1	1.00	**2.92 (1.77–4.84)**	1.26 (0.72**–**2.22)	1.43 (0.91**–**2.25)	1.14 (0.72**–**1.81)
Model 2	1.00	**2.52 (1.49–4.26)**	1.18 (0.67**–**2.10)	1.31 (0.82**–**2.10)	1.07 (0.67**–**1.71)
Model 3	1.00	**2.54 (1.50–4.24)**	1.09 (0.61**–**1.94)	1.32 (0.82**–**2.12)	1.11 (0.69**–**1.78)
**Malignancies-specific mortality**					
Model 1	1.00	1.28 (0.71**–**2.29)	1.16 (0.65**–**1.91)	0.69 (0.39**–**1.22)	0.91 (0.57**–**1.45)
Model 2	1.00	1.23 (0.64**–**2.38)	1.09 (0.62**–**1.94)	0.66 (0.36**–**1.19)	0.84 (0.53**–**1.36)
Model 3	1.00	1.16 (0.61**–**2.25)	1.02 (0.58**–**1.80)	0.63 (0.35**–**1.15)	0.86 (0.53**–**1.38)

Model 1: adjusted for age, race, gender and the length of follow-up by weighted COXPH model; Model 2: further adjusted from model 1 for BMI, CCI, HEI, medication usage, HDL-C, LDL-C, TG and TC status by weighted COXPH; Model 3: further adjusted from model 1 for BMI, CCI, HEI, medication usage, HDL-C, LDL-C, TG and TC status by weighted CRR model for cardiovascular/malignancies-specific mortality; Bold font means *p* < 0.05. CCI, Charlson Comorbidity Index; HEI, healthy eating index; BMI, body mass index; TC, total cholesterol; TG, triglyceride; HDL-C, high-density lipoprotein-cholesterol; LDL-C, low-density lipoprotein-cholesterol; COXPH, Cox proportional hazard; CRR, competing risk regression.

### Non-linear association between serum 25-hydroxy-vitamin D mortality hazard ratios

**FIGURE 3:**
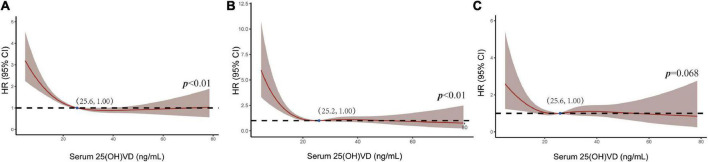
The RCS between 25(OH)VD and the mortality HRs for all-cause **(A)**, cardiovascular **(B)**, and malignancies-specific mortality **(C)**. The brown ribbon indicates the 95% CIs.

Restricted cubic spline (RCS) showed an “L-shape” association between the serum 25(OH)VD levels and all causes-specific and cardiovascular-specific mortality rates. The HRs for all causes and the cardiovascular-specific mortality significantly increased as serum 25(OH)VD decreased, at thresholds of 25.6 and 25.2 ng/mL, respectively. Above those thresholds, the HRs remained relatively stable for all mortalities. The similar association was noted between the malignancies-specific mortality HRs and serum 25(OH)VD, with the threshold below 25.6 ng/mL, but that association only approached statistical significance (*p* = 0.068) (Figure 3).

## Discussion

Vitamin D (VD) plays an important role in pathophysiology of metabolic diseases, including diabetes, hyperuricemia, HL, obesity and metabolic syndrome (20–22). However, the relationship between serum 25(OH)VD and mortality of patients with HL are still unknown and may vary depending on the patients’ clinical (23, 24), demographic (25), and lifestyle characteristics (26). To address this issue, we performed a large-scale cohort analysis of a representative adult population in the United States. Our findings indicate the serum 25(OH)VD status as an independent risk factor associated with all-cause and cardiovascular mortality in patients with HL. The findings were adjusted for known covariables, including the participants’ demographics, dietary style, as well as clinical features such as BMI, lipids status, medication usage, and comorbidities. Moreover, our study also indicated non-linear relationships between serum 25(OH)VD and the HRs of all-cause and cardiovascular mortality in patients with HL, showing a threshold in serum 25(OH)VD below which the risk for mortality exponentially increases.

Several studies have shown that higher vitamin D level was associated with decreased mortality in patients with metabolic diseases, such as type II diabetes (5) and hypertension (27). However, it is still debatable whether VD supplementation could reduce the risk of mortality in patients with cardiometabolic diseases (28–30). Some studies have shown that low VD levels are associated with worse clinical presentations in several diseases, including asthma (31), COVID-19 infection (32), and hyperuricemia (33), and that VD supplementation can improve the clinical outcomes. However, many studies also highlighted that the benefits of VD may be overestimated (17, 34). Hence, these studies suggest that VD does not have an impact on the disease itself, but the elimination of VD deficiency is what actually improves survival. Our findings also supported this view. Serum 25(OH)VD levels below 25.6 and 25.2 ng/mL had a strong impact on the increased HRs for all-cause and cardiovascular mortalities, respectively. These findings were consistent with those reported by Wan (5), Zhao (4), and Al-khalidi (35). Moreover, Jani et al. (36) performed a meta-analysis, which showed that the lower level of circulating 25(OH)VD was dose-dependently associated with the higher risks of fatal, non-fatal and recurrent cardiovascular disease, while the thresholds varied for different outcomes. Heath et al. (37) made another meta-analysis that showed an inverse relationship between circulating 25(OH)VD status and cancer-specific mortality with moderate evidence, while for cardiovascular mortality, there was weak evidence showing its association with 25(OH)VD.

As for cancer mortality, in the present study we observed also an inverse association between lower serum 25(OH)VD (below 25.6 ng/mL) and cancer mortality, but this association only approached statistical significance, which might be due to the limited cases. Additionally, although several studies have shown that lower serum 25(OH)VD were related to increased cancer mortality, not all studies confirmed that, and the associations varied depending on the type of malignancy, race, sex, season/latitude and present co-morbidities (38–43). Furthermore, the U-shaped association between VD levels and overall and cancer-mortality have been shown in some studies (44). In conclusion, more studies including different cancer types are needed to investigate the associations between serum 25(OH)VD and cancer-specific mortality.

A deficiency of VD is common in various populations due to inadequate dietary styles and lack of sunshine (45). The established cut-off values for deficiency, insufficiency, normal values, excess, and intoxication in sunny countries are <20, 20–32, 54–90, >100, and >150 ng/mL, respectively (46). However, these cut-off points are still debatable and may vary in different populations due to variations in exposure to sunshine, dietary styles, and disease incidence (47). Even though there is a general consensus is that serum 25(OH)VD below 20 ng/mL is classified as VD deficiency (48), more studies are needed to establish the optimal serum 25(OH)VD levels for individuals with various health conditions.

Considering the association between serum levels of lipid profiles and vitamin D, it is still debatable whether higher level of VD is related to decreased serum levels of LDL-C, TC, and TG and increased level of HDL-C. In the present study, elevated serum 25(OH)VD were associated with an increased level of HDL-C and TC, and lower serum 25(OH)VD were associated with higher TG and LDL-C levels. Many researchers have also reported similar results as our study (49, 50). However, elevated serum 25(OH)VD were also associated with higher level of TC in our study, which might be owing to the participants in our study were diagnosed with different types of HL, which could lead to a selection bias, since the relationship between VD levels and serum levels of lipid profiles might be different according to various individual clinical statuses (5, 51, 52). Further research is needed for investigating these relationships.

The main strength of this study is that the large population was included. The data obtained from the NHANES survey allowed us to adjust the models for various variables, including the participants’ baseline characteristics, dietary condition, comorbidities, and medication usage. However, the study has several limitations that have to be acknowledged. Although serum 25(OH)VD is a good biomarker and typically represents the VD status for nearly 2 months (46), these measurements may vary over time. However, in our study, the serum 25(OH)VD were only acquired at a single point in time. Moreover, we have not adjusted for season/altitude. Furthermore, the dietary style and sunshine exposure vary around the world, and therefore our findings cannot be generalized to other populations, and further studies are therefore recommended.

## Conclusion

The serum 25(OH)VD were identified as an independent risk factor for all-cause and cardiovascular mortalities in patients with HL in the present study. Lower serum 25(OH)VD levels were associated with a significantly higher risk for all-cause and cardiovascular mortality, at the threshold of levels lower than 25.6 and 25.2 ng/ml, respectively. However, no significant associations were found between malignancy-specific mortality and serum 25(OH)VD, even the similar trend was noted under the threshold of 25.6 ng/mL.

## Data availability statement

The raw data supporting the conclusions of this article are available in the NHANES repository (https://www.cdc.gov/nchs/nhanes/index.htm).

## Ethics statement

The studies involving human participants were reviewed and approved by the National Health and Nutrition Examination Survey, ethical review and approval and written informed consents for participation were included in NHANES databases. The patients/participants provided their written informed consent to participate in this study.

## Author contributions

XC contributed to analyzing and interpreted data. XM contributed to designing the study. JC participated in data interpretation. HY, MZ, and YW assisted to depict figures and tables. All authors contributed to the article and approved the submitted version.
